# Clinical features, proximate causes, and consequences of active convulsive epilepsy in Africa

**DOI:** 10.1111/epi.12392

**Published:** 2013-10-07

**Authors:** Symon M Kariuki, William Matuja, Albert Akpalu, Angelina Kakooza-Mwesige, Martin Chabi, Ryan G Wagner, Myles Connor, Eddie Chengo, Anthony K Ngugi, Rachael Odhiambo, Christian Bottomley, Steven White, Josemir W Sander, Brian G R Neville, Charles R J C Newton, on behalf of the SEEDS writing group

**Affiliations:** * Centre for Geographic Medicine Research Coast, Kenya Medical Research InstituteKilifi, Kenya; † Studies of Epidemiology of Epilepsy in Demographic Surveillance Systems (SEEDS)–INDEPTH NetworkAccra, Ghana; ‡ Department of Psychiatry, University of OxfordOxford, United Kingdom; § Department of Medicine, Muhimbili University College of Health ScienceDar es Salaam, Tanzania; ¶ Kintampo Health Research CentreKintampo, Ghana; # Iganga-Mayuge Health and Demographic Surveillance SystemIganga, Kampala, Uganda; ** Department of Paediatrics and Child Health, Makerere University College of Health SciencesKampala, Uganda; †† Department of Paediatrics, Ministry of Medical ServicesNairobi, Kenya; ‡‡ Medical Research Council/Wits University Rural Public Health and Health Transitions Research Unit (Agincourt), Faculty of Health Sciences, School of Public Health, University of the WitwatersrandJohannesburg, South Africa; §§ Research Support Unit, Faculty of Health Sciences, Aga Khan University (East Africa)Nairobi, Kenya; ¶¶ Department of Infectious Disease Epidemiology, Faculty of Epidemiology and Population Health, London School of Hygiene and Tropical MedicineLondon, United Kingdom; ## Department of Neurophysiology, Great Ormond Street HospitalLondon, United Kingdom; *** NIHR University College London Hospitals Biomedical Research Centre, UCL Institute of Neurology, London and Epilepsy SocietyChalfont St Peter, Bucks, United Kingdom; ††† SEIN – Stichting Epilepsie Instellingen NederlandHeemstede, The Netherlands; ‡‡‡ Neurosciences Unit, UCL Institute of Child HealthLondon, United Kingdom; §§§ Clinical Research Unit, London School of Hygiene and Tropical MedicineLondon, United Kingdom

**Keywords:** Sub-Saharan Africa, Clinical features, Active convulsive epilepsy, Comorbidity, Population-based study

## Abstract

**Purpose:**

Epilepsy is common in sub-Saharan Africa (SSA), but the clinical features and consequences are poorly characterized. Most studies are hospital-based, and few studies have compared different ecological sites in SSA. We described active convulsive epilepsy (ACE) identified in cross-sectional community-based surveys in SSA, to understand the proximate causes, features, and consequences.

**Methods:**

We performed a detailed clinical and neurophysiologic description of ACE cases identified from a community survey of 584,586 people using medical history, neurologic examination, and electroencephalography (EEG) data from five sites in Africa: South Africa; Tanzania; Uganda; Kenya; and Ghana. The cases were examined by clinicians to discover risk factors, clinical features, and consequences of epilepsy. We used logistic regression to determine the epilepsy factors associated with medical comorbidities.

**Key Findings:**

Half (51%) of the 2,170 people with ACE were children and 69% of seizures began in childhood. Focal features (EEG, seizure types, and neurologic deficits) were present in 58% of ACE cases, and these varied significantly with site. Status epilepticus occurred in 25% of people with ACE. Only 36% received antiepileptic drugs (phenobarbital was the most common drug [95%]), and the proportion varied significantly with the site. Proximate causes of ACE were adverse perinatal events (11%) for onset of seizures before 18 years; and acute encephalopathy (10%) and head injury prior to seizure onset (3%). Important comorbidities were malnutrition (15%), cognitive impairment (23%), and neurologic deficits (15%). The consequences of ACE were burns (16%), head injuries (postseizure) (1%), lack of education (43%), and being unmarried (67%) or unemployed (57%) in adults, all significantly more common than in those without epilepsy.

**Significance:**

There were significant differences in the comorbidities across sites. Focal features are common in ACE, suggesting identifiable and preventable causes. Malnutrition and cognitive and neurologic deficits are common in people with ACE and should be integrated into the management of epilepsy in this region. Consequences of epilepsy such as burns, lack of education, poor marriage prospects, and unemployment need to be addressed.


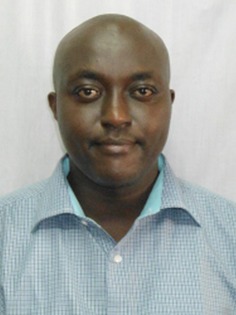


Epilepsy is a common neurologic disorder, with >85% of people with epilepsy living in low- and middle-income countries (LMICs), including sub-Saharan Africa (SSA; Ngugi et al., 2010). The increased burden of epilepsy may be related to poor health care services and increased incidence of risk factors such as central nervous system (CNS) infections (Peltola, 2001; Newton & Garcia, 2012). Epilepsy remains a neglected condition throughout the world, and we need a better understanding of the pathogenesis, management, and consequences. Many people with epilepsy in LMICs do not seek biomedical treatment for their epilepsy (Meyer et al., 2010), owing to cost or cultural beliefs (Mbuba et al., 2009, 2012). Poor adherence to antiepileptic drugs (AEDs) may contribute to poor seizure control, cognitive impairment, behavioral disorders, and excess mortality (Kariuki et al., 2012).

Epilepsy in SSA is poorly understood. Few studies have described clinical features (Tekle-Haimanot et al., 1990; Kaiser et al., 2000; Munyoki et al., 2010), and most were hospital-based and may not represent the epilepsy found in the general population, as many people with epilepsy (up to 90%) do not use medical facilities (Preux & Druet-Cabanac, 2005). The clinical features of epilepsy may vary across Africa, as the risk factors and genetic propensity may differ. One study compared these features across multiple populations in Africa and found that epilepsy is largely untreated (79%) and is associated with malnutrition (Quet et al., 2011). This study, however, compiled epilepsy data collected from previous studies that may have used different designs, and so these findings should be substantiated with a prospective multisite study using standard methodology.

Convulsive seizures are more easily recognized than nonconvulsive forms of epilepsy in community surveys. They are associated with premature mortality, greater stigma, and considerable comorbidity (Ding et al., 2006; Birbeck et al., 2007). Epilepsy in SSA is associated with medical and psychosocial consequences such as burns, unemployment, poor education, and being unmarried (Birbeck et al., 2007; Munyoki et al., 2010). Malnutrition is common (25%; Quet et al., 2011), but the relationship with epilepsy is not fully understood, as malnutrition could be either a cause (due to deficiency of micronutrients (Johnson, 2001) or a consequence of epilepsy (e.g., due to stigma; Crepin et al., 2007). It is postulated that malnutrition in epilepsy is associated with a lowered seizure threshold (Hackett & Iype, 2001), but this requires further confirmation.

We conducted a multisite study to provide a clinical description of epilepsy, to generate findings that will help plan preventative or management interventions for people with epilepsy in SSA.

## Materials and Methods

### Study sites and population

The study sites included the following health demographic surveillance systems (HDSS) as part of the International Network for the Demographic Evaluation of Populations and Their Health (INDEPTH): Agincourt, South Africa; Kintampo, Ghana; Kilifi, Kenya; Ifakara, Tanzania; and Iganga-Mayuge, Uganda, details of which are available in Table S1 and the (INDEPTH) website (http://www.indepth-network.org/).

### Identification of active convulsive epilepsy

Cases were identified in community cross-sectional surveys between August 2008 and April 2011 (Ngugi et al., 2013). The three-stage surveys screened a population of 586,584 resident at five sites (Table 1; Ngugi et al., 2012). Stage I screened for convulsive seizures using two questions to household heads about the inhabitants of the households. Stage II used additional questions with increased specificity about epilepsy to the individuals with convulsions within 1 week of stage I by epilepsy-trained field workers. The third stage required a medical history and neurologic examination to confirm the diagnosis of epilepsy. A few additional eligible cases (who had not been detected in the three-stage method) were identified following referral by clinic staff, community leaders, and following a population screening sample used to assess the sensitivity of the three-stage methodology. An assessment of cognitive impairments was based on the person's awareness of person, place, and time; and ability to follow standardized instructions during the neurologic examination. Study participants were asked about use of AEDs and history of febrile or nonfebrile seizures in the family, including parents or siblings. Electroencephalography (EEG) was performed on people with active convulsive epilepsy (ACE) using a 16 lead-channel according to the 10–20 system with hyperventilation and photic stimulation. We randomly selected community controls without epilepsy from the HDSS databases as detailed in a previous report (Ngugi et al., 2013) to allow comparison of the consequences of epilepsy.

**Table 1:** Sociodemographic characteristics and medical comorbidities of active convulsive epilepsy in five sites in Africa

Characteristics	Agincourt, South Africa (n = 331)	Ifakara, Tanzania (n = 460)	Iganga, Uganda (n = 241)	Kilifi, Kenya (n = 766)	Kintampo, Ghana (n = 372)	Total (n = 2,170)
Sociodemographics and family history						
Median age (IQR) in years	26.0 (14.0–41.0)	17.6 (11.2–30.9)	11.0 (6.0–19.0)	17.0 (9.0–27.0)	21.0 (14.0–28.0)	18.0 (11.0–30.0)
Males (%)	170 (51.4)	216/456 (47.0)	129 (53.5)	395/765 (51.6)	203/339 (54.6)	1,113 (51.3)
Age at onset of seizures (IQR) in years	12.7 (3.4–27.0)	10.1 (2.4–17.40)	2.0 (0.6–5.9)	3.0 (1.0–12.2)	9.0 (3.0–15.0)	5.3 (1.3–15.0)
Family history of seizures (%)	13 (3.9)	98 (21.3)	35 (14.5)	183 (23.9)	66 (17.7)	395 (18.2)
Parents with history of seizures (%)	5 (1.5)	13 (2.8)	8 (3.3)	32 (4.2)	7 (1.9)	65 (3.0)
Siblings with history of seizures (%)	15 (4.5)	39 (8.5)	40 (16.6)	46 (6.0)	79 (21.2)	219 (10.1)
Family history of febrile seizures (%)	4 (1.2)	4 (0.9)	48 (19.9)	102 (13.3)	26 (7.0)	184 (8.5)
Comorbidities of active convulsive epilepsy						
Malnutrition (%)	31 (9.4)	61 (13.3)	50 (20.8)	142 (18.5)	36 (9.7)	320 (14.8)
Neurologic deficits (%)	69 (18.1)	38 (8.3)	43 (17.8)	135 (17.6)	43 (11.6)	319 (14.7)
Focal neurologic deficits (%)	69 (18.1)	23 (5.0)	41 (17.0)	128 (16.7)	40 (10.8)	301/2,152 (13.9)
Cognitive impairment (%)	87 (26.3)	56 (12.2)	43 (17.8)	208 (27.2)	100 (26.9)	494 (22.8)

### Definition of epilepsy and classification

Epilepsy, defined as ≥2 unprovoked seizures (ILAE, 1993), was classified as active if seizures had occurred in the previous 12 months, a criterion used locally (Ministry of Health Kenya, 2002). Seizures were classified by a pediatric neurologist (CN), together with site-specific neurologists, and disagreements were resolved by consensus through a neurologic panel. We classified seizures as focal, generalized, or others using a classification system recently devised for epidemiologic studies: Thurman et al. (2011); focal epilepsy was defined as focal seizure semiology and focal epileptiform discharges on EEG, whereas generalized epilepsy was defined as generalized seizure semiology and generalized epileptiform discharges on EEG. Seizure frequency was categorized into daily (at least one each day), weekly (at least one a week), monthly (at least one a month), and yearly (at least one a year). Status epilepticus was defined as seizures lasting for 30 min or intermittent seizures for a period of 30 min as timed by a watch or, for those without watches, events such as boiling a pot of maize, news bulletin on radio, or milking a cow, all which take about 30 min. Children were defined as those aged between 0 and 18 years and adults those older than 18 years.

### Determination of nutritional status and head circumference

Malnutrition was defined as height for age z-scores below −2 for those aged 0–10 years, body mass index (BMI; weight/height^2^) value in the lower 5th percentile for those aged 11–19 years, and BMI value <18.5 for those >19 years old (Quet et al., 2011). A small head circumference (a z-score below −2) was computed for those up to 3 years for whom World Health Organization (WHO) reference curves are available (Ivanovic et al., 2004). The z-scores were computed using a Stata command *zanthro* (2000 CDC Growth Reference in the U.S.A.; College Station, TX, U.S.A.).

### Definition of proximate causes of ACE

Proximate causes were those conditions known from previous studies to be directly linked or proximate to the development of epilepsy. Causes were defined as follows: Adverse perinatal events (newborn baby not breathing or crying immediately after birth and seizure onset <18 years of age), head injuries before seizure onset (by a day or more), sickle cell disease (a lifetime risk of neurologic disorders assumed; Zeldine, 1978), and cardiovascular complications, for example, stroke if seizures occurred after the onset of hemiparesis. Alcohol intake and acute encephalopathy (coma related to an acute malarial, viral, or bacterial illness) were considered proximate causes regardless of age.

### Statistical analysis

The data were double entered and verified in MySQL Version 5 open source database (Oracle Corporation, Redwood Shores, CA, U.S.A.). All analyses were performed using Stata (version 11). Student's *t*-test was used to compare age and age-at-onset of seizures (data appeared normally distributed) between groups. One-way analysis of variance (ANOVA) was used to compare these continuous variables across the five sites and six age-groups (0–5, 6–12, 13–18, 19–28, 29–49, and 50+ years). Pearson's chi-square test was used to compare categorical variables between groups. Odds ratios (ORs) were calculated using multivariable logistic regression to quantify associations between epilepsy factors (age-at-onset of seizures, status epilepticus, focal epilepsy, abnormal EEG, frequent seizures) and AED use, medical/neurologic comorbidities, and consequences of ACE. The ORs computed in the multivariable analysis were adjusted for potential confounding effects of age, sex, and site, a priori. Univariate associations with a p-value < 0.25 were used to build a multivariate model using a backward elimination method. The score test for variables per unit increase in age were tested using Mantel-Haenszel methods.

### Ethical approval

Permission was approved by the local institutional ethical committees and from the ethics committee at UCL Institute of Child Health, London, United Kingdom. Informed consent was obtained for each person.

## Results

### Demographic characteristics of people with ACE

In the five sites, 2,170 people fulfilled the criteria for ACE, of whom 1,711 were identified from the three-stage survey. A further 459 were assessed after voluntarily presenting to clinic. Of the 2,170 cases, 729 (33.4%) had focal EEG and focal seizures suggestive of focal epilepsy. There were 1,101/2,170 (51%) children and 51% males with ACE (Table 1). The median (interquartile range [IRQ]) age in years was 18.0 (11.0–30.0), and females were older (median age 19.0 vs. 17.0, p = 0.023). There was a significant age difference across the five sites, with median age lowest in Iganga and highest in Agincourt (p = 0.0001; Table 1).

### Age at onset of seizures

The median (IQR) age at onset of seizure across the five sites was 5.3 years (1.33–15.05) with no sex differences (p = 0.121). The age at onset of seizures varied across the five sites (p < 0.001). The median age at seizure onset was lowest in Iganga (2.0 years) and highest in Agincourt (12.7 years; Table 1). Seizures began before 12 years of age for 1,277/2,170 (58.9%) people with ACE. The total number of people with ACE and the proportion that began at specific ages of onset are shown (Fig. S1), and seizures began in childhood in 505/1,069 (47.2%) adults with ACE. The median age of onset did not differ between people with the focal and the generalized ACE type (p = 0.908), but appeared earlier in those with a history of impaired consciousness, that is, acute encephalopathy (median age onset 13.0 vs. 19.0 years, p < 0.001).

### Family history of seizures

A family history of any seizures (regardless of degree of family relationship or association with fever) was reported in 395/2,170 (18.2%) people with ACE, with significant differences across sites (p < 0.001), being highest in Kilifi (23.9%; Table 1). The prevalence of a family history of seizures was similar in those with focal and generalized epilepsy (p = 0.658). Family history of febrile seizures was reported by 184/2,170 (8.5%) overall, and differed according to site (p < 0.001), being highest in Iganga (19.9%). A history of seizures among siblings occurred in 219/2,170 (10.1%), with significant differences across the sites (p < 0.001), being highest in Kintampo (21.2%). A history of seizures among parents was reported in 65/2,170 (3.0%), with no significant differences across the sites (p = 0.095), but was highest in Kilifi (4.2%).

### Seizure semiology and electroencephalography

All primarily generalized seizures occurred in 1,075/2,170 (49.5%) people with ACE (Table 2), and the proportions were similar in children and adults (p = 0.957). Focal seizures (977/2,170 [45.0%]) were similar in children and adults (p = 0.100), with significant differences across sites (p < 0.0001); highest in Kilifi (64.6%) and lowest in Ifakara (31.4%; Table 2).

**Table 2:** Seizure types, seizure frequency, and treatment of convulsive active epilepsy in five sites in Africa

Characteristics	Agincourt, South Africa (n = 331)	Ifakara, Tanzania (n = 460)	Iganga, Uganda (n = 241)	Kilifi, Kenya (n = 766)	Kintampo, Ghana (n = 372)	Total (n = 2,170)
Seizure types according to Thurman et al. (2011)						
All primarily generalized seizures	198 (59.8)	231 (50.2)	161 (66.8)	249 (32.5)	236 (63.4)	1,075 (49.5)
Generalized tonic–clonic seizures (%)	164 (49.6)	206 (44.9)	143 (59.3)	210 (27.5)	220 (59.1)	943 (43.5)
Generalized other convulsive seizures (%)	17 (5.1)	3/459 (0.7)	8/240 (3.3)	20/760 (2.6)	9/371 (2.4)	57/2,160 (2.6)
Generalized absence seizures (%)	29 (8.8)	10 (2.2)	6 (2.5)	25 (3.3)	19 (5.1)	89 (4.1)
Generalized unspecified seizures (%)	6 (1.8)	24 (5.2)	8 (3.3)	6 (0.8)	6 (1.6)	50 (2.3)
Focal seizure types						
All focal seizures	134 (40.5)	144 (31.3)	78 (32.4)	494 (64.5)	126 (34.1)	977 (45.0)
Focal becoming secondarily generalized seizures (%)	122 (36.9)	107 (23.3)	61 (25.3)	236 (30.8)	107 (28.8)	633 (29.2)
Focal convulsive seizures	5 (1.5)	15 (3.3)	4 (1.7)	184 (24.0)	3 (0.8)	211 (9.7)
Focal dyscognitive seizures	14 (4.2)	11 (2.4)	13 (5.4)	92 (12.0)	22 (5.9)	152 (7.0)
Focal sensory seizures	0	6 (1.3)	0	10 (1.3)	1 (0.3)	17 (0.8)
Focal unspecified seizures	0	16 (3.5)	2 (0.8)	28 (3.7)	0	46 (2.1)
Other unspecified seizures	5 (1.5)	8 (1.7)	1 (0.4)	9 (1.2)	0	23 (1.1)
Other convulsive seizures	0	0	2 (0.8)	26 (3.4)	4 (1.1)	32 (1.5)
Impaired consciousness	0	1 (0.2)	0	1 (0.1)	0	2 (0.1)
Seizure frequency						
Daily (%)	24 (7.3)	58 (12.6)	32 (13.3)	98 (12.8)	56 (15.1)	268 (12.4)
Weekly (%)	16 (4.8)	29 (6.3)	34 (14.1)	84 (11.0)	40 (10.8)	203 (9.4)
Monthly (%)	142 (42.9)	267 (58.0)	68 (28.2)	254 (33.2)	161 (43.3)	892 (41.1)
Yearly (%)	149 (45.0)	106 (23.0)	107 (44.4)	330 (43.1)	115 (30.9)	807 (37.2)
Status epilepticus						
Status epilepticus (%)	78/250 (31.2)	16/340 (4.7)	90/224 (40.2)	254/722 (35.2)	29/346 (8.4)	467/1,882 (24.8)
Febrile status epilepticus (%)	24/250 (9.6)	3/340 (0.9)	33/224 (14.7)	186/722 (25.8)	5/346 (1.5)	251/1,882 (13.3)
Antiepileptic drugs (AEDs)						
Self-reported AED use (%)	171 (51.7)	193 (42.0)	52 (21.6)	322 (42.0)	48 (12.9)	786 (37.6)

AED, antiepileptic drug.

Unclassifiable seizures occurred in 57/2,170 (2.6%), and were more common in children than adults (39/1,101 [3.5%] vs. 17/1,069 [1.6%]; p = 0.007). There were significant differences across the sites (p < 0.0001), being most common in Kilifi (4.7%) and Ifakara (2.0%; Table 2).

Overall, EEG was performed in 1,426/2,170 (65.7%) people with ACE, in 721/1,075 (67.0%) of those with generalized seizure semiology, and in 669/977 (71.5%) of those with focal seizure semiology. An abnormal EEG was found in 377/721 (52.3%) of people with ACE and a generalized seizure semiology. Focal EEG features were present in 210/721 (29.1%) of generalized seizures. The focal EEG features in those who had generalized seizure semiology were localized most often to temporofrontal 153/210 (72.9%) and parietooccipital 105/210 (50.0%) lobes, with 48/210 (22.9%) occurring in both sides of the brain. Centrotemporal epileptiform discharges were present in 165/548 (30.1%) children aged 0–15 years, with significant differences across the sites (p < 0.001).

**Figure 1:**
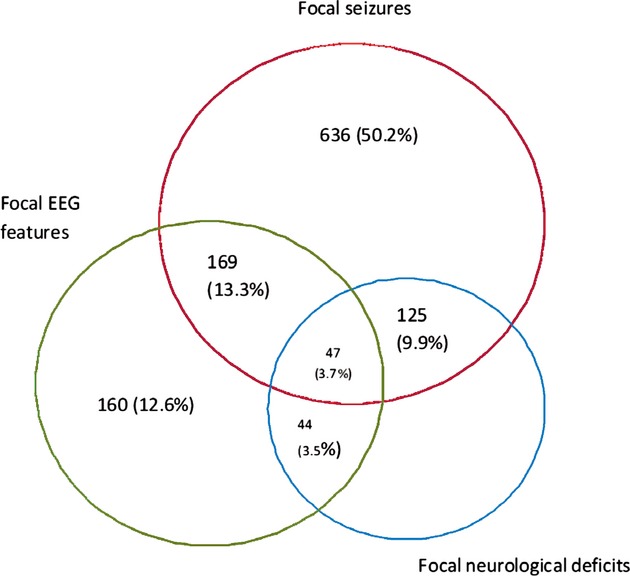
The overlap of focal seizures, focal electroencephalography (EEG) features, and focal neurologic deficits across the five sites. About two thirds of people with active convulsive epilepsy had at least one of the three focal features documented.

An abnormal EEG was found in 374/699 (55.9%) of people with ACE who had a focal seizure semiology, with 217/374 (58.0%) of the abnormal EEG being focal EEG features. The highest concordance for focal EEG features and focal semiology was seen in Agincourt (60/92 [65.2%]) and Ifakara (26/65 [40.0%]), but Kendall's tau-b statistics suggests that this may be due to chance (Kendall's τ-b = 0.068 for Agincourt and 0.070 for Ifakara). Focal seizures overlapped substantially with focal EEG and focal neurologic deficits (n = 1,267/2,170 [58.4%]; Fig. 1).

### Seizure frequency

Overall 1,363/2,170 (62.8%) people with ACE experienced frequent seizures on a daily, weekly, or monthly basis, with the remainder (37.2%) experiencing less frequent seizures on a yearly basis. Of the 1,363 with very frequent seizures, seizures occurred daily in 20%, weekly in 15%, and monthly in 65%, with significant differences across the five sites (p < 0.001; Table 2). Daily seizures occurred more frequently in children than in adults (148/641 [23.1%] vs. 120/722 [16.6%]; p = 0.003) and in those with an abnormal EEG than with a normal EEG (117/544 [21.5%] vs. 65/360 [18.1%]; p = 0.033), but in similar proportions in those with focal and generalized ACE (p = 0.310).

### Status epilepticus

Information about status epilepticus was obtained from 1,882/2,170 (86.7%) people with ACE. Of these 1,882 people with ACE, status epilepticus was reported by 24.8%, with significant differences across the sites (p < 0.001; Table 2). Status epilepticus was associated with a febrile illness in 251/1,864 (13.5%) people with ACE, with 18 people not knowing whether it occurred with febrile illness or not. Status epilepticus was more common in those with history of acute encephalopathy than in those without (198/208 [95.2%] vs. 269/1,674 [16.1%]; p < 0.001), and in those using AEDs than in those not using (198/690 [28.7%] vs. 269/1,992 [22.6%]; p = 0.003).

### Antiepileptic drug use

Overall, 786/2,170 (36.2%) of people with ACE reported using AEDs, with significant variation between sites (p < 0.001), being highest in Agincourt (51.7%; Table 2). Of these 786 on AEDs, 744/786 (94.7%) recalled using phenobarbital, 314 (40.0%) carbamazepine, 227 (28.9%) phenytoin, 220 (28.0%) diazepam, and 26 (3.3%) sodium valproate. Adults used AEDs more than children did (467/1,069 [43.7%]) versus (319/1,101 [29.0%]; p < 0.001). AEDS were used equally by male and female patients (p = 0.305), but were used more in focal (330/729 [45.3%]) than in generalized epilepsy (456/1,141 [31.6%]; p < 0.001). The factors independently associated with AED use were focal seizures (adjusted odds ratio [aOR] 1.77; 95% CI 1.26–2.49; p = 0.001), abnormal EEG (aOR 1.66; 95% CI 1.17–2.34; p = 0.004) and high seizure frequency (aOR 1.29; 95% CI 1.05–1.58; p = 0.014).

### Proximate causes of ACE

Adverse perinatal events were reported in 119/1,101 (10.8%) children, and differed across five sites (p < 0.001; Table 3). They occurred in similar proportions between generalized and focal epilepsy (p = 0.089). Adverse perinatal events and age at seizure onset were not related (univariate OR 0.98; 95% CI 0.94–1.03; p = 0.462). Microcephaly was weakly associated with perinatal complications among the children with ACE (p = 0.064). A small head circumference was present in 10/101 (9.9%) of children aged 0–3 years (whose WHO reference charts were available), with similar proportions across sites (p = 0.082).

**Table 3:** Proximate causes of active convulsive epilepsy in five sites in Africa

Characteristics	Agincourt, South Africa (n = 331)	Ifakara, Tanzania (n = 460)	Iganga, Uganda (n = 241)	Kilifi, Kenya (n = 766)	Kintampo, Ghana (n = 372)	Total (n = 2,170)
Adverse perinatal events (%)^a^	11 (3.3)	22 (4.8)	11 (4.6)	34 (4.4)	43 (11.6)	121 (5.6)
Acute encephalopathy (%)	6 (1.8)	4 (0.9)	32 (13.3)	162 (21.2)	5 (1.3)	209 (9.6)
All head injuries (%)	35 (10.6)	20 (4.4)	11 (4.6)	93 (12.1)	92 (24.7)	251 (11.6)
Head injury before seizure onset (%)	10 (3.0)	8 (1.7)	0	27 (3.5)	9 (2.4)	54 (2.5)
Sickle cell disease (%)	2 (0.6)	1 (0.2)	0	2 (0.3)	2 (0.5)	7 (0.3)
Hypertension (%)	25 (7.6)	2 (0.4)	1 (0.4)	8 (1.0)	1 (0.3)	37 (1.7)
Stroke (%)	11 (3.3)	1 (0.2)	1 (0.4)	3 (0.4)	2 (0.6)	18 (0.8)
Drinks alcohol	35 (10.6)	43 (9.4)	9 (3.7)	44 (5.7)	65 (17.5)	196 (9.0)

a Adverse perinatal events were considered reliable only in those whose seizures began before 18 years.

Head injuries, before and after onset of seizures, were reported in 251/2,131 (11.8%), with differences across sites (p < 0.001; Table 3). They were more common in male (158/1,073 [14.8%]) than in female patients (80/1,004 [8.0%]; p < 0.001). The 251 head injuries were associated with loss of consciousness in 148 (59.0%) and hospitalization in 77 (30.7%). Head injuries occurred before seizures in 54/2,170 (2.5%) and were similar in focal and generalized epilepsy (p = 0.620). Stroke was reported in 18/2,170 (0.8%) people with ACE, with significant differences across sites (p < 0.001), and between focal (11/729 [1.5%]) and generalized epilepsy (7/1,441 [0.5%]; p = 0.013).

Sickle cell disease occurred in 7/2,170 (0.3%) people with ACE, being similar across the sites (Fisher's exact p = 0.705), and in focal and generalized epilepsy (Fisher's exact p = 1.000; Table 3). The odds of stroke increased in older age-group (OR 1.82; 95% CI 1.32–2.54; p < 0.001), with stroke being reported in 8/454 (1.8%) whose seizures began after 35 years.

Acute encephalopathy was documented in 209/2,170 (9.6%), with significant differences across the sites (Fisher's exact p < 0.001). Encephalopathy varied between children (149/1,101 [13.5%]) and adults (60/1,069 [5.6%]; p < 0.001) and between focal (86/729 [11.8%]) and generalized ACE (123/1,441 [8.5%]; p = 0.015). Alcohol intake was reported in 196/2,170 (9.0%), with significant differences across sites (p < 0.001). Expected alcohol use was more common in adults 193/1,069 (18.1%) than in children (3/1,101); p < 0.001, but was similar in those with focal and those with generalized epilepsy (p = 0.060).

### Comorbidities of active convulsive epilepsy

Univariate associations for the comorbidities outlined below are shown in Table S2.

#### Malnutrition

Malnutrition was present in 320/2,170 (14.8%) people with ACE, with significant differences across the sites (p < 0.001; Table 1). The prevalence of malnutrition did not differ between people with focal and generalized ACE (p = 0.653), or between consumers and nonconsumers of cassava (p = 0.579) or between children below 3 years with and without microcephaly (p = 0.661). Seizure frequency was independently associated with malnutrition (aOR 1.34; 95% CI 1.11–1.61; p = 0.002).

#### Neurologic deficits

Neurologic deficits were present in 319/2,170 (14.7%) of people with ACE, differed across sites (p < 0.001), and 301/319 (94.5%) were unilateral/focal (Table 1). The prevalence was similar in children and adults (p = 0.670) and in both sexes (p = 0.243), but it was higher in focal (163/720 [22.6%]) than in generalized epilepsy (155/1,409 [11.0%]; p < 0.001). Neurologic deficits were independently associated with age at onset of seizures (aOR 0.96: 95% CI 0.94–0.99, p = 0.009), abnormal EEG (aOR 4.12; 95% CI 2.32–7.33, p < 0.001), frequent seizures (defined as those occurring either daily or weekly; aOR 1.75; 95% CI 1.35–2.27; p < 0.001), and status epilepticus (aOR 1.78; 95% CI 1.10–2.89; p = 0.019).

#### Cognitive impairment

Cognitive impairment occurred in 494/2,170 (22.8%) and differed across sites (p < 0.001), being more common in Kintampo (100/372 [26.9%]; Table 1). Impairment did not differ between children and adults (p = 0.071), was more common in male (281/1,113 [25.3%]) than in female individuals (213/1,057 [20.2%]; p = 0.005), and occurred more in focal (225/729 [30.9%]) than generalized epilepsy 269/1,441 (18.7%; p < 0.001). Cognitive impairment was independently associated with abnormal EEG (aOR 3.17; 95% CI 2.03–4.94; p < 0.001), AED use (aOR 2.14; 95% CI 1.40–3.25; p < 0.001), frequent seizures (aOR 1.34; 95% CI 1.06–1.71; p = 0.016), status epilepticus (aOR 1.91; 95% CI 1.22–2.98; p = 0.004), and age at onset of seizures (aOR 0.95; 95% CI 0.92–0.97; p = 0.002).

### Consequences of active convulsive epilepsy

#### Burns

Burn marks were seen in 347/2,170 (16.0%) people, and was greater than in age-matched controls (123/1,988 [6.2%]; p < 0.001). The occurrence differed across sites (p < 0.001), being most frequent in Kilifi (20.4%; Table 4). Burns were more common in adults (230/1,069 [21.5%]) than in children (117/1,101 [10.6%]; p < 0.001), in females (204/1,057 [19.3%]) than in males (143/1,113 [12.9%]; p < 0.001), and in focal (135/729 [18.5%]) than in generalized epilepsy (212/1,441 [14.7%]; p = 0.022). AED use was independently associated with burns (OR 1.51; 95% CI 1.05–2.24; p = 0.040).

**Table 4:** Consequences of active convulsive epilepsy in five sites in Africa

Characteristics	Agincourt, South Africa (n = 331)	Ifakara, Tanzania (n = 460)	Iganga, Uganda (n = 241)	Kilifi, Kenya (n = 766)	Kintampo, Ghana (n = 372)	Total (n = 2,170)
Burns (%)	49 (14.8)	66 (14.4)	18 (7.5)	156 (20.4)	58 (15.6)	347 (16.0)
Head injury after seizure onset (%)	18 (5.4)	1 (0.2)	0	2 (0.3)	4 (1.1)	25/1,905 (1.2)
Nonattendance at school (%)	96 (29.0)	194 (42.2)	111 (46.1)	383 (50.0)	154 (41.4)	938 (43.2)
Unemployed adults (%)^a^	211 (63.8)	57 (12.4)	42 (17.4)	225 (29.4)	111 (29.8)	646 (29.8)
Unmarried adults (%)	224 (67.7)	174 (37.8)	72 (29.9)	297 (38.8)	201 (54.0)	968 (44.6)

a Adults were defined as those aged older than 18 years.

#### Head injury after seizure onset

Head injuries after seizures onset occurred in 25/2,170 (1.2%) and differed across the sites (p < 0.001; Table 4). The occurrence was more common in males (18/1,113 [1.6%]) than in females (7/1,057 [0.7%]; p = 0.037), and in focal (14/729 [1.9%]) than in generalized epilepsy (11/1,441 [0.8%]; p = 0.017). There were insufficient data to build a multivariate model.

#### Education

Lack of education was reported in 938/2,170 (43.2%) and was greater than in age-matched controls (484/1,993 [24.3%]; p < 0.001). It varied between sites (p < 0.001), being most common in Kilifi (50.0%; Table 4). It was more common in children (543/1,101 [49.3%]) than in adults (395/1,069 [37.0%]; p < 0.001) and in females (487/1,057 [46.1%]) than in males (451/1,113 [40.5%]; p = 0.011), but was similar in focal and generalized epilepsy (p = 0.203). Lack of education was independently associated with frequent seizures (aOR 1.26; 95% CI 1.02–1.55; p = 0.029) and younger age at onset of seizures (aOR 0.97; 95% CI 0.96–0.99; p = 0.004).

#### Unemployment

About 611/1,010 (57.2%) adults were unemployed (lack of an economic activity), greater than age-matched controls (343/906 [37.9%]), p < 0.001. Unemployment in adults varied between sites (p < 0.001), being more common in Agincourt (64%; Table 4) and in those with focal (235/351 [67.0%]) than those with generalized epilepsy (376/718 [52.4%]), p < 0.001, with no differences between sex (p = 0.200). Status epilepticus was independently associated with unemployment in adults (OR 2.33; 95% CI 1.23–4.42; p = 0.009).

#### Marriage

Adults with ACE were less likely to be married than adult controls (716/1,069 [67.0%] vs. 354/922 [38.4%]; p < 0.001). The proportion of unmarried adults differed across the sites (p < 0.001), being highest in Agincourt (68%; Table 4). Unmarried adults were similar in both sexes (p = 0.569) and in generalized and focal epilepsy (p = 0.170). AED use was independently associated with being unmarried (OR 1.67; 95% CI 1.10–2.54; p = 0.015).

## Discussion

This study describes possible causes, clinical features, and consequences of ACE in five populations in SSA. We chose to screen for ACE only because it is easy to identify in a resource poor setting, is associated with a poorer outcome, and is the criteria for starting AED in most African countries. More than half of those identified with epilepsy were children, and in most adults (47%) seizures started in childhood. Focal features were common (60%), considering this study included convulsive epilepsy only, and this proportion varied with site. The most common identified proximate cause was adverse perinatal events (12%). Thirty-eight percent of people were using AEDs, but use varied with site. Comorbidities were common, particularly cognitive impairments (23%). Psychosocial and medical consequences were significantly more common in those with epilepsy than in community controls. These clinical features differed across sites probably because of difference in risk factors, which would require site-specific interventions.

### Relationship between age and epilepsy

Overall half of identified cases occurred in children, and seizures started in childhood in more than two thirds of people with ACE. A history of acute encephalopathy was higher in children. Onset varied with site, earliest in Iganga, perhaps because this site is holoendemic for malaria (Pullan et al., 2010), and later in Agincourt, which had the highest proportion of chronic adult illnesses such as hypertension and stroke.

### Focal features are common

Focal epilepsy is common (60%), despite our study design, which included only convulsive seizures, as these are easier to identify in a resource-poor setting. A considerable proportion of convulsive seizures in SSA may have a focal origin but rapidly generalize (Gwer et al., 2012). Focal seizures are more common in structural lesions (Gibbs et al., 1993) and are more likely associated with preventable causes, which in African settings could include cerebral malaria, bacterial meningitis, and neurocysticercosis. Focal seizures have a poor neurobehavioral outcome in Africa (Kariuki et al., 2012) and were associated with greater cognitive impairment and neurologic deficits.

### Proximate causes of epilepsy

A family history of seizures, regardless of degree, was present in a fifth of people and febrile seizures in siblings in a 10th. Febrile seizures in close relatives were similar for generalized and focal epilepsy, but further genetic studies are required.

More than a 10th of people with ACE had a history of perinatal complications. Case–control studies from Africa have consistently identified perinatal risk factors (Burton et al., 2012; Ngugi et al., 2013). In one study, small head circumference was associated with perinatal complications (Burton et al., 2012), although microcephaly may be determined by genetic or nutritional factors (Ivanovic et al., 2004; Gegios et al., 2010). Encephalopathies were common, and studies have reported an association between falciparum malaria encephalopathy and epilepsy (Birbeck et al., 2010). Early life insults can modify the acute encephalopathy and increase the overall risk of epilepsy (Holmes, 2000). Alcohol intake, stroke, and sickle cell could cause epilepsy, particularly in adults (Freedland & McMicken, 1993; Paradowski & Zagrajek, 2005), but the latter two were reported rarely, probably because of poor recall and lack of specialized diagnostic services.

### Seizure frequency and status epilepticus

Most people experienced monthly or weekly seizures, but in 20% they occurred daily. Frequent seizures were associated with younger age and abnormal EEG. Status epilepticus had occurred in 25%, similar to the findings of other studies (DeLorenzo et al., 1995). The association between young age and frequent seizures probably occurred because children in this study were less likely to be taking AEDs than adults were. An abnormal EEG may be a marker of underlying neurologic impairment, often associated with increased seizure frequency (Moran et al., 2004). Acute encephalopathy and younger age were associated with status epilepticus in children with epilepsy, as would be expected (Sadarangani et al., 2008).

### Medical comorbidities of epilepsy

Malnutrition was common, occurring in up to 25% of children younger than 10 years, a proportion comparable to other studies of epilepsy from Africa (Quet et al., 2011). However, the relationship between malnutrition and epilepsy is uncertain (Crepin et al., 2007), but better nutritional status may improve outcome. Further studies to demonstrate the improvement of nutrition with epilepsy control are required. Cognitive and neurologic impairments were common, and both were associated with high untreated seizure frequency, thus proper treatment and adherence might improve outcome. The association between AED use and cognitive functioning may occur because people with severe epilepsy take AEDs, since phenobarbital use did not affect cognition in a Chinese study (Banu et al., 2007).

### Consequences of epilepsy

Epilepsy had detrimental effects, all of which were common. Burns and lack of education were more common in female patients, which could be associated with their domestic responsibilities (Munyoki et al., 2010) and sociocultural restrictions. AED use was associated with burns, suggesting that burns are common in severe epilepsy. These consequences strongly affect the quality of life of people with ACE, sometimes more than the seizures themselves (Baca et al., 2011). Most consequences were associated with prolonged and frequent seizures and could be alleviated with proper treatment. Injuries and burn scars may exacerbate epilepsy-associated stigma, and should be addressed through counseling to increase marriage, employment, and attendance at schools (Baker, 2002).

### Strengths and limitations

A similar methodology was used across the five sites to provide standard neurologic data for planning interventions. Seizures were classified using an international standardized criteria (Thurman et al., 2011), but this was limited by lack of neuroimaging services. The cultural issues in each country may affect the identification (and classification) of epilepsy. Details on history of some clinical features such as perinatal events and status epilepticus may have been subject to recall bias. Cognition was not assessed with neuropsychological tests, but was based on standardized clinical assessment.

## Conclusion

Focal features and onset in childhood are common in people with ACE in Africa, suggesting identifiable underlying causes that may be prevented or treated. The clinical features differ across sites, probably because of difference in underlying risk factors, which would require site-specific preventive and treatment interventions. Comorbidities were common in ACE, most importantly malnutrition and cognitive impairment, calling for programs of care that address these problems, particularly in children.

## Appendix 1

The SEEDS writing group members: *Agincourt Health and Demographic Surveillance System (HDSS), South Africa:* Ryan Wagner, Rhian Twine, Myles Connor, F. Xavier Gómez Olivé, Mark Collinson, Kathleen Kahn, Stephen Tollman; *Ifakara HDSS, Tanzania:* Honratio Masanja, Alexander Mathew (deceased); *Iganga-Mayuge HDSS, Uganda:* Angelina Kakooza, George Pariyo, Stefan Peterson, Donald Ndyomughenyi; *Kilifi HDSS, Kenya**:*** Symon M. Kariuki, Anthony K. Ngugi, Rachael Odhiambo, Eddie Chengo, Martin Chabi, Evasius Bauni, Gathoni Kamuyu, Victor Mung'ala Odera (deceased), James O. Mageto, Charles R. Newton; *Kintampo HDSS, Ghana:* Ken Ae-Ngibise, Bright Akpalu, Albert Akpalu, Francis Agbokey, Patrick Adjei, Seth Owusu-Agyei; *London School of Hygiene and Tropical Medicine, United Kingdom:* Christian Bottomley, Immo Kleinschmidt; *King's College London, United Kingdom:* Victor C. K. Doku; *Swiss Tropical Institute, Switzerland:* Peter Odermatt; *University College London, United Kingdom:* Brian Neville, Josemir W. Sander, Steve White; *National Institutes of Health, U.S.A.:* Thomas Nutman; *Centers for Disease Control and Prevention, U.S.A.:* Patricia Wilkins, John Noh.

## Supporting Information

**Table S1.** Description of the five demographic sites participating in the study. **Table S2.** The epilepsy factors associated with comorbidities, consequences and treatment of active convulsive epilepsy in the univariable analysis across five sites in Africa.

**Figure S1.** The total number of people with ACE for each age group and the proportion that had seizures begin at that age group. About 69% of people with ACE had their seizures begin in childhood.
