# Supplementary material for: Clinical features, proximate causes, and consequences of active convulsive epilepsy in Africa
Source: Epilepsia. 2013 Oct 7;55(1):76–85. doi: 10.1111/epi.12392 (PMC4074306; doi:10.1111/epi.12392)
Supplement: Supplementary file 1 [file epi0055-0076-SD1.doc]

**Supplementary table 1: Description of the five** demographic sites participating in the study

| **Characteristics** | **Agincourt, South Africa** | **Ifakara, Tanzania** | **Iganga, Uganda** | **Kilifi, Kenya** | **Kintampo, Ghana** |
| --- | --- | --- | --- | --- | --- |
|  | Located in a semi-arid area of the northeast of South Africa adjacent to Mozambique border. Has an area of 420 km^2^. The crude mortality ratio is 22/1,000. Hosts studies on HIV, cerebrovascular diseases mortality rates and health systems. | Located in rural Southern Tanzania. The crude mortality ratio is 7.6/1,000. Studies on malaria, health systems and maternal/child health problems have been conducted | Located near the shores of Lake Victoria and established in 2005 under the auspice of Makerere University. It conducts vital data on births, deaths and other socially useful data and hosts studies on malaria vaccine trials, TB surveillance system and a newborn survival study. | Located along the Kenya coast in a malaria-endemic area. Literacy levels and utilization of health services are low. Has a population of 300,000 people and the death rate in under 5 year old children is 65/1,000. Conducts studies on malaria, epilepsy and other infectious diseases. | Located in central Ghana and is formed of North and South Kintampo districts. Covers an area of 7162 km^2^ and the crude mortality ratio is 7.8/1,000. Most people are subsistence farmers and fishermen. It conducts studies on malaria, and mental health disorders. |
| **Population in first survey** | 82,795 | 93,423 | 64,143 | 232,176 | 113,784 |
| **Number with active convulsive epilepsy** | 331 | 460 | 241 | 766 | 372 |

**Supplementary table 2: The epilepsy factors associated with comorbidities, consequences and treatment of active convulsive epilepsy in the univariable analysis across fives sites in Africa**

|  | Agincourt, South Africa | | | Ifakara, Tanzania | | | | Iganga, Uganda | | | Kilifi, Kenya | | | Kintampo, Ghana | | | All sites | |
| --- | --- | --- | --- | --- | --- | --- | --- | --- | --- | --- | --- | --- | --- | --- | --- | --- | --- | --- |
| Characteristic | OR (95%CI) | P-Value | | OR (95%CI) | P-Value | | | OR (95%CI) | P-Value | | OR (95%CI) | P-Value | | OR (95%CI) | P-Value | | OR (95%CI) | P-Value |
| **AED use versus non-AEDs use** | | | | | | | | | | | | | | | | | | |
| Age at onset of seizures | 1.00 (0.99-1.02) | 0.239 | | 0.98 (0.96-1.00) | 0.112 | | | 1.01 (0.98-1.05) | 0.399 | | 0.99 (0.98-1.00) | 0.958 | | 1.01 (0.98-1.03) | | 0.637 | 1.00 (0.99-1.01) | 0.081 |
| Focal seizures | 1.57 (0.98-2.51) | 0.062 | | 1.06 (0.70-1.60) | 0.778 | | | 2.30 (1.22-4.33) | 0.010 | | 1.94 (1.42-2.65) | <0.001 | | 2.38 (1.29-4.40) | | 0.006 | 1.75 (1.47-2.10) | <0.001 |
| Abnormal EEG | 1.46 (0.82-2.55) | 0.197 | | 2.17 (1.23-3.84) | 0.008 | | | 1.98 (0.91-4.33) | 0.087 | | 2.01 (1.40-2.91) | <0.001 | | 0.93 (0.50-1.75) | | 0.822 | 1.76 (1.42-2.20) | <0.001 |
| Status epilepticus | 0.87 (0.49-1.53) | 0.629 | | 1.37 (0.48-3.87) | 0.553 | | | 1.67 (0.88-3.18) | 0.116 | | 1.12 (0.82-1.53) | 0.464 | | 2.87 (1.12-7.36) | | 0.028 | 1.35 (1.09-1.68) | 0.006 |
| Seizure frequency | 1.21 (0.93-2.74) | 0.642 | | 1.53 (0.93-2.51) | 0.092 | | | 2.07 (0.93-4.59) | 0.075 | | 0.65 (0.44-0.95) | 0.027 | | 5.12 (2.50-10.50) | | <0.001 | 1.08 (0.95-1.24) | 0.251 |
| **Malnutrition versus non-malnutrition** | | | | | | | | | | | | | | | | | | |
| Age at onset of seizures | 1.03 (1.00-1.05) | 0.013 | | 0.98 (0.95-1.01) | 0.225 | | | 1.00 (0.99-1.04) | 0.257 | | 1.00 (0.99-1.01) | 0.976 | | 1.04 (1.00-1.06) | 0.008 | | 1.00 (0.99-1.01) | 0.520 |
| Focal seizures | 0.58 (0.26-1.30) | 0.186 | | 0.75 (0.41-1.38) | 0.361 | | | 0.97 (0.50-1.90) | 0.934 | | 0.74 (0.51-1.07) | 0.112 | | 0.81 (0.38-1.70) | 0.572 | | 0.86 (0.67-1.10) | 0.233 |
| Abnormal EEG | 1.05 (0.44-2.51) | 0.962 | | 1.26 (0.52-3.06) | 0.606 | | | 0.97 (0.42-2.20) | 0.933 | | 1.12 (0.70-1.80) | 0.635 | | 1.20 (0.58-2.48) | 0.622 | | 1.11 (0.82-1.51) | 0.489 |
| Status epilepticus | 0.22 (0.05-0.98) | 0.048 | | 0.41 (0.53-3.20) | 0.396 | | | 0.99 (0.52-1.90) | 0.982 | | 0.99 (0.66-1.47) | 0.945 | | - | - | | 0.94 (0.70-1.27) | 0.698 |
| AEDs use | 1.36 (0.58-3.19) | 0.479 | | 1.03 (0.59-1.79) | 0.908 | | | 1.47 (0.70-3.06) | 0.298 | | 0.49 (0.33-0.72) | <0.0001 | | 0.81 (0.27-2.41) | 0.704 | | 0.76 (0.59-0.98) | 0.035 |
| Seizure frequency | 1.20 (0.63-2.26) | 0.580 | | 1.00 (0.66-1.50) | 0.984 | | | 0.97 (0.59-1.59) | 0.904 | | 1.40 (1.05-1.85) | 0.020 | | 1.45 (0.90-2.33) | 0.130 | | 1.28 (1.07-1.52) | 0.007 |
| **Neurological deficits versus absence of neurological deficits** | | | | | | | | | | | | | | | | | | |
| Age at onset of seizures | 1.02 (0.99-1.03) | 0.112 | | 1.00 (0.93-1.09) | | 0.824 | | 2.54 (1.52-4.23) | | 0.001 | 1.17 (0.96-1.43) | | 0.128 | 1.03 (1.00-1.06) | | 0.072 | 0.98 (0.97-0.99) | <0.001 |
| Focal seizures | 1.84 (1.04-3.23) | 0.035 | | 1.29 (0.65-2.58) | | 0.467 | | 1.90 (0.97-3.72) | | 0.063 | 1.53 (1.01-2.31) | | 0.042 | 1.16 (0.60-2.25) | | 0.656 | 1.65 (1.30-2.10) | <0.001 |
| Abnormal EEG | 2.07 (1.02-4.72) | 0.045 | | 9.10 (2.07-40.02) | | 0.003 | | 7.92 (2.50-25.00) | | <0.001 | 2.58 (1.52-4.38) | | <0.001 | 5.68 (2.41-13.23) | | <0.001 | 3.50 (2.49-4.93) | <0.001 |
| Status epilepticus | 1.38 (0.71-2.66) | 0.343 | | 3.64 (1.10-12.07) | | 0.034 | | 1.32 (0.66-2.64) | | 0.430 | 2.47 (1.68-3.62) | | <0.001 | 3.14 (1.15-8.56) | | 0.025 | 2.39 (1.84-3.11) | <0.001 |
| Frequent seizures | 1.18 (0.71-1.94) | 0.523 | | 2.52 (1.67-3.80) | | <0.001 | | 2.00 (1.24-3.23) | | 0.005 | 1.66 (1.28-2.17) | | <0.001 | 1.18 (0.80-1.76) | | 0.408 | 1.69 (1.43-1.99) | <0.001 |
| AED use | 2.08 (1.09-4.00) | 0.027 | | 1.30 (0.64-2.66) | | 0.464 | | 3.09 (1.51-6.32) | | 0.002 | 0.92 (0.63-1.35) | | 0.673 | 1.66 (0.72-3.85) | | 0.238 | 1.36 (1.06-1.74) | 0.015 |
| **Cognitive impairment versus absence of cognitive impairment** | | | | | | | | | | | | | | | | | | |
| Age at onset of seizures | 0.97 (0.95-0.99) | 0.001 | | 0.95 (0.91-0.99) | | 0.025 | | 0.99 (0.95-1.03) | | 0.585 | 0.95 (0.93-0.97) | <0.001 | | 0.98 (0.95-1.00) | | 0.063 | 0.93 (0.95-0.97) | <0.001 |
| Focal seizures | 1.48 (0.90-2.44) | 0.120 | | 1.47 (0.83-2.63) | | 0.187 | | 1.93 (0.98-3.80) | | 0.057 | 1.19 (0.85-1.67) | 0.309 | | 1.64 (1.02-2.65) | | 0.042 | 1.98 (1.53-2.57) | <0.001 |
| Abnormal EEG | 2.53 (1.33-4.80) | 0.005 | | 5.28 (1.76-15.85) | | 0.003 | | 5.89 (2.02-17.16) | | 0.001 | 3.88 (2.41-6.24) | <0.001 | | 3.05 (1.82-5.11) | | <0.001 | 3.44 (2.60-4.55) | <0.001 |
| Status epilepticus | 1.17 (0.65-2.13) | 0.600 | | 3.03 (1.00-9.16) | | 0.049 | | 1.31 (0.66-2.61) | | 0.445 | 2.56 (1.83-3.57) | <0.001 | | 1.70 (0.71-4.07) | | 0.235 | 2.00 (1.59-2.52) | <0.001 |
| Frequent seizures | 1.76 (1.14-2.72) | 0.011 | | 2.18 (1.54-3.08) | | <0.001 | | 1.82 (1.13-2.94) | | 0.014 | 1.59 (1.25-2.01) | <0.001 | | 0.87 (0.62-1.20) | | 0.392 | 1.56 (1.27-1.91) | <0.001 |
| AED use | 3.02 (1.67-5.47) | <0.001 | | 2.11 (1.16-3.82) | | 0.014 | | 3.07 (1.50-6.29) | | 0.002 | 1.90 (1.38-2.62) | <0.001 | | 1.10 (0.56-2.15) | | 0.790 | 1.80 (1.46-2.21) | <0.001 |
| **Burns versus no burns** | | | | | | | | | | | | | | | | | | |
| Age at onset of seizures | 1.00 (0.98-1.02) | | 0.801 | 0.99 (0.96-1.02) | | | 0.531 | 0.98 (0.92-1.05) | | 0.582 | 0.99 (0.98-1.01) | 0.474 | | 1.01 (0.98-1.03) | | 0.703 | 0.99 (0.98-1.01) | 0.693 |
| Focal seizures | 1.78 (0.96-3.28) | | 0.066 | 0.74 (0.41-1.33) | | | 0.312 | 1.75 (0.66-4.63) | | 0.259 | 1.45 (0.99-2.12) | 0.059 | | 1.00 (0.55-1.80) | | 0.991 | 1.41 (1.12-1.78) | <0.001 |
| Abnormal EEG | 0.90 (0.42-1.96) | | 0.797 | 0.81 (0.35-1.71) | | | 0.579 | 1.50 (0.41-5.43) | | 0.537 | 1.57 (0.98-2.52) | 0.063 | | 1.31 (0.73-2.36) | | 0.371 | 1.30 (0.97-1.74) | 0.079 |
| Status epilepticus | 0.42 (0.18-1.00) | | 0.051 | 1.37 (0.37-5.01) | | | 0.639 | 0.82 (0.29-2.30) | | 0.703 | 0.93 (0.64-1.36) | 0.709 | | 0.22 (0.03-1.68) | | 0.144 | 0.84 (0.63-1.13) | 0.245 |
| Frequent seizures | 0.87 (0.50-1.51) | | 0.625 | 1.64 (1.17-2.30) | | | 0.004 | 2.32 (1.22-4.42) | | 0.010 | 1.01 (0.77-1.32) | 0.950 | | 0.73 (0.46-1.15) | | 0.171 | 1.13 (0.95-1.33) | 0.160 |
| AED use | 3.38 (1.56-7.33) | | 0.002 | 2.45 (1.40-4.30) | | | 0.002 | 1.85 (0.66-5.19) | | 0.244 | 1.82 (1.27-2.60) | 0.001 | | 0.58 (0.22-1.55) | | 0.280 | 1.86 (1.47-2.35) | <0.001 |
| **Being unmarried versus being married** | | | | | | | | | | | | | | | | | | |
| Age at onset of seizures | 1.12 (1.01-1.25) | | 0.028 | 1.04 (0.97-1.13) | | | 0.272 | 0.96 (0.84-1.08) | | 0.477 | 1.01 (0.95-1.08) | | 0.682 | 1.00 (0.90-1.10) | | 0.985 | 1.03 (0.99-1.07) | 0.051 |
| Focal seizures | 1.68 (0.66-4.29) | | 0.279 | 2.00 (1.02-3.93) | | | 0.044 | 1.13 (0.46-2.77) | | 0.792 | 1.13 (0.65-1.97) | | 0.675 | 0.41 (0.13-1.30) | | 0.131 | 1.01 (0.75-1.37) | 0.942 |
| Abnormal EEG | 0.64 (0.18-2.34) | | 0.504 | 1.63 (0.61-4.38) | | | 0.334 | 0.98 (0.33-2.92) | | 0.967 | 1.32 (0.67-2.59) | | 0.421 | 1.90 (0.69-5.26) | | 0.217 | 1.38 (0.94-2.05) | 0.104 |
| Status epilepticus | 1.72 (0.94-3.15) | | 0.078 | 0.94 (0.19-4.64) | | | 0.942 | 0.61 (0.25-1.51) | | 0.286 | 1.03 (0.60-1.77) | | 0.918 | 1.26 (0.25-6.45) | | 0.778 | 1.04 (0.84-1.28) | 0.736 |
| Frequent seizures | 0.98 (0.37-2.58) | | 0.969 | 1.11 (0.70-1.76) | | | 0.666 | 1.80 (0.84-3.85) | | 0.131 | 1.12 (0.75-1.170) | | 0.575 | 1.21 (0.68-2.17) | | 0.521 | 1.08 (0.86-1.37) | 0.513 |
| AED use | 1.73 (0.69-4.31) | | 0.241 | 1.61 (0.81-3.19) | | | 0.175 | 1.05 (0.37-3.05) | | 0.917 | 1.37 (0.80-2.35) | | 0.259 | 0.98 (0.26-3.72) | | 0.971 | 1.42 (1.03-1.97) | 0.032 |
| **Unemployment versus employment** | | | | | | | | | | | | | | | | | | |
| Age at onset of seizures | 1.18 (0.96-1.46) | | 0.118 | 1.12 (0.95-1.33) | | | 0.186 | 0.99 (0.93-1.06) | | 0.834 | 0.96 (0.92-1.02) | | 0.175 | 1.02 (0.93-1.13) | | 0.634 | 0.96 (0.93-1.99) | 0.007 |
| Focal seizures | 1.02 (0.25-4.19) | | 0.976 | - | | | - | 0.90 (0.43-1.85) | | 0.773 | 0.91 (0.59-1.40) | | 659 | 0.67 (0.25-1.83) | | 437 | 1.10 (0.85-1.42) | 0.479 |
| Abnormal EEG | - | | - | - | | | - | 1.05 (0.45-2.47) | | 0.910 | 0.81 (0.46-1.41) | | 0.453 | 0.64 (0.25-1.65) | | 0.360 | 0.75 (0.54-1.05) | 0.091 |
| Status epilepticus | 2.07 (0.57-7.52) | | 0.269 | - | | | - | 0.63 (0.32-1.25) | | 0.187 | 1.11 (0.71-1.73) | | 0.646 | 1.05 (0.21-5.27) | | 0.950 | 2.40 (1.67-3.45) | <0.001 |
| Frequent seizures | 2.12 (0.31-14.62) | | 0.446 | 1.06 (0.30-3.74) | | | 0.928 | 1.59 (0.89-2.91) | | 0.135 | 1.24 (0.87-1.75) | | 0.236 | 1.74 (0.99-3.05) | | 0.054 | 1.46 (1.19-1.80) | <0.001 |
| AED use | 1.26 (0.30-5.30) | | 0.749 | 1.16 (0.19-7.15) | | | 0.870 | 1.59 (0.64-3.98) | | 0.321 | 0.99 (0.63-1.55) | | 0.964 | 0.57 (0.12-2.69) | | 0.479 | 1.03 (0.77-1.38) | 0.830 |
| **Non-school attendance versus school attendance** | | | | | | | | | | | | | | | | | | |
| Age at onset of seizures | 0.99 (0.97-1.00) | | 0.103 | 0.91 (0.88-0.94) | | | <0.001 | 0.98 (0.96-1.01) | | 0.214 | 1.00 (0.99-1.01) | | 0.722 | 0.98 (0.96-1.00) | | 0.113 | 0.98 (0.97-0.99) | <0.001 |
| Focal seizures | 0.72 (0.44-1.18) | | 0.191 | 1.18 (0.79-1.76) | | | 0.415 | 0.93 (0.54-1.62) | | 0.804 | 0.81 (0.60-1.09) | | 0.161 | 0.80 (0.51-1.24) | | 0.316 | 0.98 (0.82-1.16) | 0.781 |
| Abnormal EEG | 1.37 (0.74-2.50) | | 0.317 | 3.99 (2.20-7.26) | | | <0.001 | 1.52 (0.78-2.98) | | 0.219 | 1.38 (0.97-1.98) | | 0.074 | 1.04 (0.67-1.61) | | 0.852 | 1.52 (1.23-1.89) | <0.001 |
| Status epilepticus | 1.14 (0.64-2.04) | | 0.649 | 9.80 (2.19-43.83) | | | <0.001 | 0.72 (0.42-1.25) | | 0.241 | 1.34 (0.98-1.83) | | 0.067 | 0.90 (0.38-2.15) | | 0.821 | 1.29 (1.05-1.60) | 0.017 |
| Frequent seizures | 1.57 (1.03-2.40) | | 0.036 | 1.52 (1.15-2.01) | | | 0.004 | 1.19 (0.78-1.82) | | 0.421 | 1.42 (1.12-1.80) | | 0.004 | 0.85 (0.63-1.15) | | 0.297 | 1.34 (1.17-1.53) | <0.001 |
| AED use | 1.35 (0.81-2.25) | | 0.248 | 1.02 (0.69-1.50) | | | 0.925 | 0.99 (0.53-1.84) | | 0.978 | 0.79 (0.59-1.05) | | 0.103 | 1.16 (0.63-2.13) | | 0.641 | 0.91 (0.76-1.09) | 0.292 |
