# Supplementary figures and images for: Clinical features, proximate causes, and consequences of active convulsive epilepsy in Africa
Source: Epilepsia. 2013 Oct 7;55(1):76–85. doi: 10.1111/epi.12392 (PMC4074306; doi:10.1111/epi.12392)

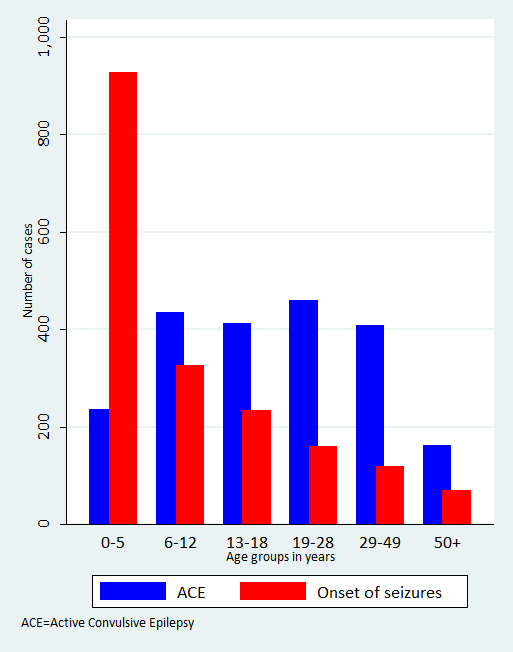

Supplement: Supplementary file 2 — Figure S1. The total number of people with ACE for each age group and the proportion that had seizures begin at that age group. About 69% of people with ACE had their seizures begin in childhood. [file epi0055-0076-SD2.doc]
