# Supplementary material for: Clinical features, proximate causes, and consequences of active convulsive epilepsy in Africa
Source: Epilepsia. 2013 Oct 7;55(1):76–85. doi: 10.1111/epi.12392 (PMC4074306; doi:10.1111/epi.12392)
Supplement: Supplementary file 3 — Figure S1. The total number of people with ACE for each age group and the proportion that had seizures begin at that age group. About 69% of people with ACE had their seizures begin in childhood. [file epi0055-0076-SD3.pptx]

## Slide 1
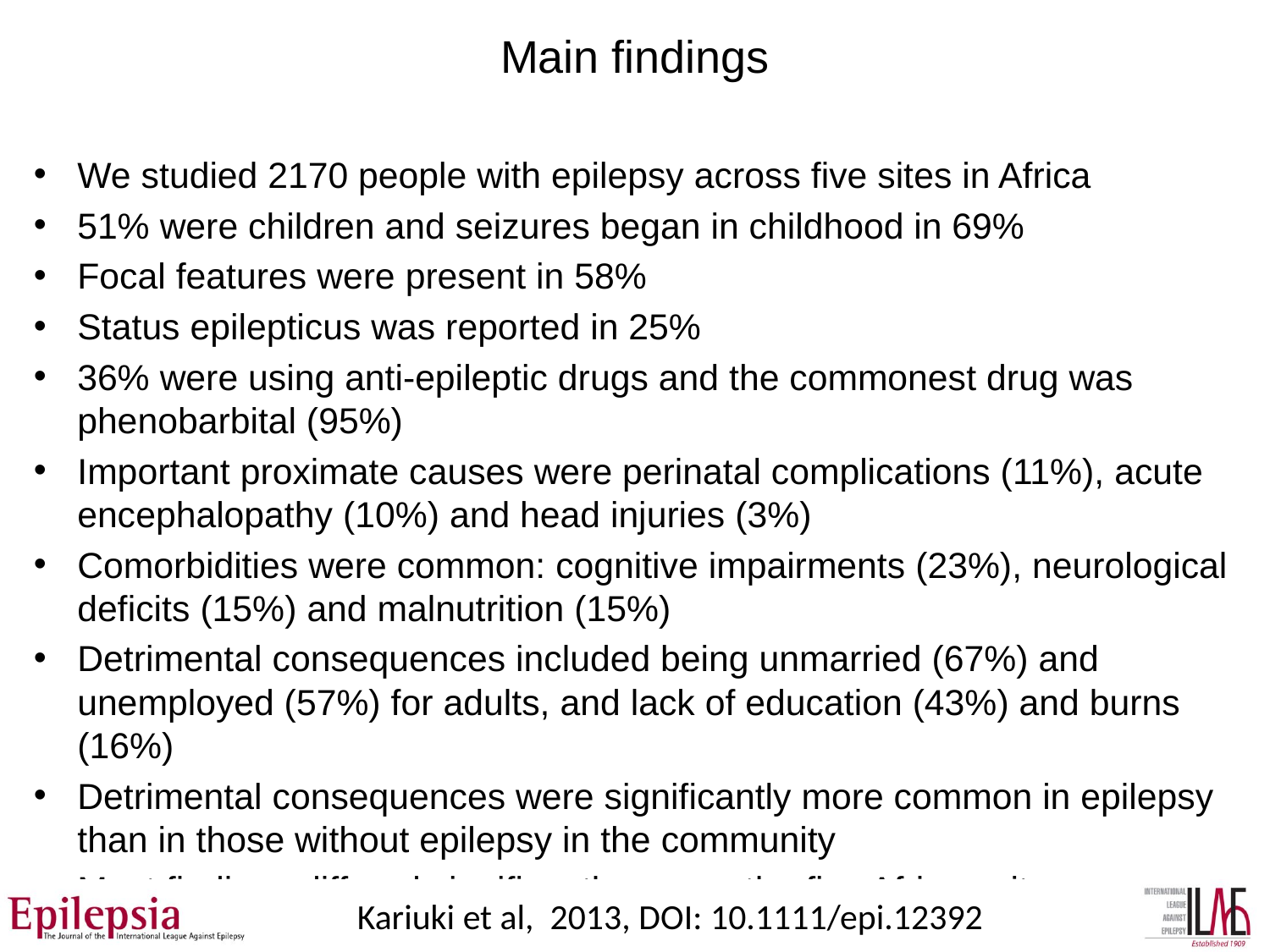

# Main findings
We studied 2170 people with epilepsy across five sites in Africa
51% were children and seizures began in childhood in 69%
Focal features were present in 58%
Status epilepticus was reported in 25%
36% were using anti-epileptic drugs and the commonest drug was phenobarbital (95%)
Important proximate causes were perinatal complications (11%), acute encephalopathy (10%) and head injuries (3%)
Comorbidities were common: cognitive impairments (23%), neurological deficits (15%) and malnutrition (15%)
Detrimental consequences included being unmarried (67%) and unemployed (57%) for adults, and lack of education (43%) and burns (16%)
Detrimental consequences were significantly more common in epilepsy than in those without epilepsy in the community
Most findings differed significantly across the five African sites
Kariuki et al, 2013, DOI: 10.1111/epi.12392
